# Statistical power in clinical trials of interventions for mood, anxiety, and psychotic disorders

**DOI:** 10.1017/S0033291722001362

**Published:** 2023-07

**Authors:** Ymkje Anna de Vries, Robert A. Schoevers, Julian P. T. Higgins, Marcus R. Munafò, Jojanneke A. Bastiaansen

**Affiliations:** 1 Department of Developmental Psychology, University of Groningen, Groningen, the Netherlands; 2 Interdisciplinary Center Psychopathology and Emotion Regulation, University Medical Center Groningen, University of Groningen, Groningen, the Netherlands; 3 Department of Psychiatry, University of Groningen, University Medical Center Groningen, Groningen, the Netherlands; 4 University of Groningen, Research School of Behavioural and Cognitive Neurosciences (BCN), Groningen, the Netherlands; 5 Population Health Sciences, Bristol Medical School, University of Bristol, Bristol, UK; 6 National Institute for Health Research Applied Research Collaboration West (ARC West) at University Hospitals Bristol and Weston NHS Foundation Trust, Bristol, UK; 7 National Institute for Health Research Bristol Biomedical Research Centre, University Hospitals Bristol and Weston NHS Foundation Trust and University of Bristol, Bristol, UK; 8 Medical Research Council Integrative Epidemiology Unit at the University of Bristol, Bristol, UK; 9 School of Psychological Science, University of Bristol, Bristol, UK; 10 Department of Education and Research, Friesland Mental Health Care Services, Leeuwarden, the Netherlands

**Keywords:** Anxiety disorders, clinical trials, complementary and alternative medicine, mood disorders, pharmacotherapy, psychotherapy, psychotic disorders, statistical power

## Abstract

**Background:**

Previous research has suggested that statistical power is suboptimal in many biomedical disciplines, but it is unclear whether power is better in trials for particular interventions, disorders, or outcome types. We therefore performed a detailed examination of power in trials of psychotherapy, pharmacotherapy, and complementary and alternative medicine (CAM) for mood, anxiety, and psychotic disorders.

**Methods:**

We extracted data from the Cochrane Database of Systematic Reviews (Mental Health). We focused on continuous efficacy outcomes and estimated power to detect predetermined effect sizes (standardized mean difference [SMD] = 0.20–0.80, primary SMD = 0.40) and meta-analytic effect sizes (ES_MA_). We performed meta-regression to estimate the influence of including underpowered studies in meta-analyses.

**Results:**

We included 256 reviews with 10 686 meta-analyses and 47 384 studies. Statistical power for continuous efficacy outcomes was very low across intervention and disorder types (overall median [IQR] power for SMD = 0.40: 0.32 [0.19–0.54]; for ES_MA_: 0.23 [0.09–0.58]), only reaching conventionally acceptable levels (80%) for SMD = 0.80. Median power to detect the ES_MA_ was higher in treatment-as-usual (TAU)/waitlist-controlled (0.49–0.63) or placebo-controlled (0.12–0.38) trials than in trials comparing active treatments (0.07–0.13). Adequately-powered studies produced smaller effect sizes than underpowered studies (*B* = −0.06, *p* ⩽ 0.001).

**Conclusions:**

Power to detect both predetermined and meta-analytic effect sizes in psychiatric trials was low across all interventions and disorders examined. Consistent with the presence of reporting bias, underpowered studies produced larger effect sizes than adequately-powered studies. These results emphasize the need to increase sample sizes and to reduce reporting bias against studies reporting null results to improve the reliability of the published literature.

## Introduction

Mental disorders are responsible for a large proportion of the global disease burden (Whiteford et al., 2013). Effective treatment options are, however, available – mainly various forms of pharmacotherapy and psychotherapy (Huhn et al., 2014), although some complementary and alternative medicine (CAM) treatments (e.g. mindfulness) also appear to be effective for some disorders (Asher et al., 2017; Kuyken et al., 2016). Consistent with the ideals of evidence-based medicine (EBM), treatment efficacy is supported by randomized controlled trials (RCTs), the gold standard for high-quality evidence. However, there has been increasing concern that the evidence base that EBM depends on is distorted. The efficacy of antidepressants and antipsychotics, for instance, has been inflated by reporting bias (de Vries et al., 2018; Roest et al., 2015; Turner, Knoepflmacher, & Shapley, 2012; Turner, Matthews, Linardatos, Tell, & Rosenthal, 2008), and the same is probably true for psychotherapy (de Vries et al., 2018; Driessen, Hollon, Bockting, Cuijpers, & Turner, 2015). Problems in trial design can also lead to stacking the deck in favor of a treatment (Heres et al., 2006; Leichsenring et al., 2017) or to difficulty generalizing results to clinical practice (Lorenzo-Luaces, Zimmerman, & Cuijpers, 2018). Here, we focus on one particular problem in trial design, namely inadequate statistical power.

Statistical power is the probability of detecting an effect of a specific size if that effect is actually present. The threshold for adequate power is conventionally set at 80% (Cohen, 1992). Inadequate statistical power not only increases the likelihood of false negatives, but also the likelihood that statistically significant effects represent false-positive findings (Button et al., 2013). The problem of underpowered trials can in principle be resolved through meta-analysis: by combining underpowered studies, a well-powered meta-analysis yields a precise estimate (Guyatt, Mills, & Elbourne, 2008). However, the problem of low power is more pernicious when combined with reporting bias, which is ubiquitous (Song et al., 2010). While underpowered studies are as likely to yield an *underestimate* of the true effect size as they are to yield an *overestimate*, reporting bias filters out (statistically non-significant) underestimates. This may result in a literature dominated by false-positives and inflated effect sizes.

Low power to detect relevant effect sizes has previously been demonstrated for studies in neuroscience (Button et al., 2013), biomedicine (Dumas-Mallet, Button, Boraud, Gonon, & Munafò, 2017), and the social sciences (Smaldino & McElreath, 2016). An examination of the Cochrane Database of Systematic Reviews (CDSR) by Turner et al. found that the median power to detect a relative risk reduction of 30% was only 14% in mental health trials (comparable with 13% for medicine in general). Furthermore, effect sizes were reduced by 12–15% when only adequately-powered studies were considered (Turner, Bird, & Higgins, 2013).

So far, no study has specifically focused on the mental health field. Although it is to be expected that power will be lower than recommended in this field as well, important questions remain. For instance, Turner et al. only included binary outcomes, even though the primary outcome in psychiatric trials is usually continuous [e.g. decrease in symptoms (Cuijpers, Li, Hofmann, & Andersson, 2010a; Roest et al., 2015; Turner et al., 2012, 2008)]. Examining only binary outcomes, for which trials were not powered, could result in a lower estimate of power than for continuous outcomes. It is therefore possible that the situation is not quite as bad as suggested by this work. Furthermore, Turner et al. only examined the power to detect the meta-analysis-specific effect size across all trials, regardless of medical specialty or intervention type. This may be important because effect sizes vary widely. Comparing antidepressants with placebo, for instance, the standardized mean difference (SMD) is around 0.3 (Roest et al., 2015; Turner et al., 2008), while the SMD for psychotherapy is around 0.9 when compared to waitlist, but much lower when compared to more active control conditions (Cuijpers, van Straten, Bohlmeijer, Hollon, & Andersson, 2010b). As statistical power primarily depends on sample size and effect size, using the same effect size across disorders, interventions, and comparators could lead to an underestimate or overestimate of power for interventions that are actually markedly more or less effective than the chosen effect size. There is some preliminary evidence that this might be the case, as a study of psychotherapy trials for depression reported that the average power to detect the meta-analytic effect size was much better, at 49% (Flint, Cuijpers, Horder, Koole, & Munafò, 2015). Moreover, pharmacotherapy trials take place within an entirely different context (e.g. often funded by industry and performed in response to regulatory requirements) than trials of psychotherapy or CAM (e.g. usually performed by academic centers with little outside oversight). The same is true for different disorders, as academic fields tend to be rather siloed and may have their own traditions, with especially little overlap between researchers working on psychotic disorders and those working on mood or anxiety disorders.

In this study, therefore, we performed a detailed examination of statistical power to detect both predetermined and meta-analysis-specific effect sizes in trials of psychotherapy, pharmacotherapy, and CAM for mood, anxiety, and psychotic disorders, the three major classes of mental disorders included in the Cochrane Collaboration's Mental Health section. We focused on continuous efficacy outcomes, but also examined other outcomes (binary efficacy and safety). We also examined whether statistical power is increasing over time. Finally, we examined whether the inclusion of underpowered studies in meta-analyses results in inflated effect sizes. This fine-grained comparison of statistical power can provide clinicians and researchers with a better sense of where the problem of low power is most acute and hence with starting points for improvements.

## Methods

### Data source and selection

This study was registered after we received the data, but before performing any analyses (osf.io/hgaec). With permission from the Cochrane Collaboration, we received an export of currently published systematic reviews of interventions in the Mental Health area in RevMan (RM5) format in October 2017 and an updated dataset in March 2022. We extracted the following information from each review: review title, comparison, outcome, subgroup, names of group 1 and group 2, effect direction, study names, type of effect measure (e.g. SMD), effect size with confidence interval and standard error (if available), number of events in each group (for binary outcomes), and sample size in each group. Each combination of comparison, outcome, and subgroup made up a single meta-analysis.

Reviews were categorized by topic and intervention by YV (checked by JB). We categorized each review into mood disorders, anxiety disorders, and psychotic disorders. Reviews that did not fit one of these categories (e.g. interventions for aggression) or fit multiple categories were excluded, unless individual meta-analyses could be assigned to a specific category. We also assigned each review to pharmacotherapy (PHT), psychotherapy (PST), or CAM [defined based on a topic list provided for the Cochrane Collaboration (Wieland, Manheimer, & Berman, 2005)]. Reviews that did not clearly fit one of these categories were excluded. Reviews or meta-analyses that investigated combination PHT and PST were assigned to PST if the comparator was PHT, to PHT if the comparator was PST, or excluded if the comparator was treatment as usual.

We excluded meta-analyses that only included a single study; that were not analyzable because the event rate was 0, the outcome was time-to-event, or the sample size was clearly mistaken (0 or 1 in each group); or that used unusual control interventions (i.e. that did not match pharmacotherapy, psychotherapy, CAM, placebo, treatment as usual, waitlist, or a combination of these). Meta-analyses were assigned to one of four categories by YV based on the description of the outcome (with any unclear outcomes checked by JB) and the effect measure (odds ratio [OR]/risk ratio/risk difference *v.* (standardized) mean difference): (1) continuous efficacy outcome (e.g. symptom questionnaires), (2) binary efficacy outcome (e.g. relapse), (3) continuous safety outcome (e.g. weight gain), or (4) binary safety outcome (e.g. occurrence of nausea). We chose the continuous efficacy measure as our primary outcome, as change in disorder symptoms is most commonly used as the primary outcome in psychiatry. However, most studies provided multiple continuous efficacy outcomes, and no information was available about which of these outcomes (if any) was the original primary trial outcome, so we included all available trial outcomes.

### Effect size and power calculations

We first re-calculated meta-analyses using a mean difference, risk difference, or risk ratio as an outcome to use the SMD or OR instead. Hence, we mean a standardized effect size (SMD or OR) whenever we refer to effect size. Random-effects meta-analysis was performed using restricted maximum likelihood estimation (REML) via the *rma* command from the *metafor* package (2.4–0) in R (4.0.0). We multiplied SMDs by −1 and took the inverse of ORs where necessary to ensure that positive SMDs or ORs greater than 1 favored the intervention group. For active *v.* active comparisons (e.g. antidepressant *v.* another antidepressant), we used the absolute effect size or the inverse of the OR (if OR < 1), as experimental and comparator conditions can be seen as interchangeable.

We estimated the power of each study to detect predetermined small to large effect sizes (SMD = 0.20, 0.40, 0.60 or 0.80, or the roughly equivalent OR = 1.5, 2.0, 3.0, and 4.5, using the formula log(OR) = SMD × *π*/sqrt(3) (Da Costa et al., 2012) and rounded to the nearest 0.5). We set SMD = 0.40 as the primary effect size in our study (i.e. the effect size of greatest interest), as this is close to the mean effect size for psychiatric treatments in general (Huhn et al., 2014; Leucht, Hierl, Kissling, Dold, & Davis, 2012). We also estimated each study's power to detect the effect size of the meta-analysis it was included in (ES_MA_), as a proxy for the true effectiveness/safety of an intervention. We calculated the power for each study using the *pwr.t2n.test* command for continuous outcomes and the *pwr.2p2n.test* command for binary outcomes [*pwr* package (1.3–0)]. To illustrate, the formula to determine power for a two-sided, two-sample *t* test is:
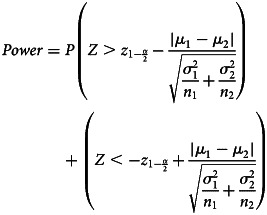


This formula illustrates that power is dependent upon *α* (customarily set at 0.05), sample sizes (*n*_1_ and *n*_2_), the difference in means between the groups (*μ*_1_ − *μ*_2_), and variability in the outcome (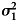 and 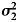). The latter are essentially taken together in a standardized effect size, which incorporates both the difference in means and variability.

To examine trends in power to detect SMD = 0.40 over time, we plotted median power against the publication year.

### Meta-regression analysis of adequate power

Following Turner et al. (Turner et al., 2013), we investigated the impact of underpowered studies on the estimated effect size of continuous efficacy outcomes. We selected meta-analyses that included ⩾5 studies, of which ⩾2 were adequately powered (⩾80%) and ⩾1 was underpowered. For each group of studies in a meta-analysis, we fit a random-effects meta-regression model with a term for ‘adequate power’. Subsequently, we used random-effects meta-analysis to summarize the effect of adequate power across meta-analyses.

### Sensitivity analyses

We performed several planned sensitivity analyses for the continuous efficacy outcome. First, we calculated the power to detect the ES_avg_, defined as the meta-analytic average effect size of all meta-analyses for each combination of outcome (efficacy *v.* safety), outcome type (binary *v.* continuous), experimental group, and comparator group. While the ES_MA_ is a specific, but potentially very noisy, proxy for the ‘true’ effect size of a specific intervention for a specific outcome (e.g. paroxetine *v.* placebo for depressive symptoms), the ES_avg_ is more stable but less specific because it is aggregated across similar interventions and outcomes (e.g. pharmacotherapy *v.* placebo for any continuous efficacy outcome). Second, we recalculated the power to detect the ES_MA_ after excluding meta-analyses with very small effect sizes (ES_MA_ < 0.2). Third, we recalculated power using the effect size of the largest trial in each meta-analysis, to account for possible publication bias. Finally, because studies could be included in multiple meta-analyses, we recalculated power while only including each study once.

## Results

### Data selection and characteristics of included reviews

**Fig. 1.**
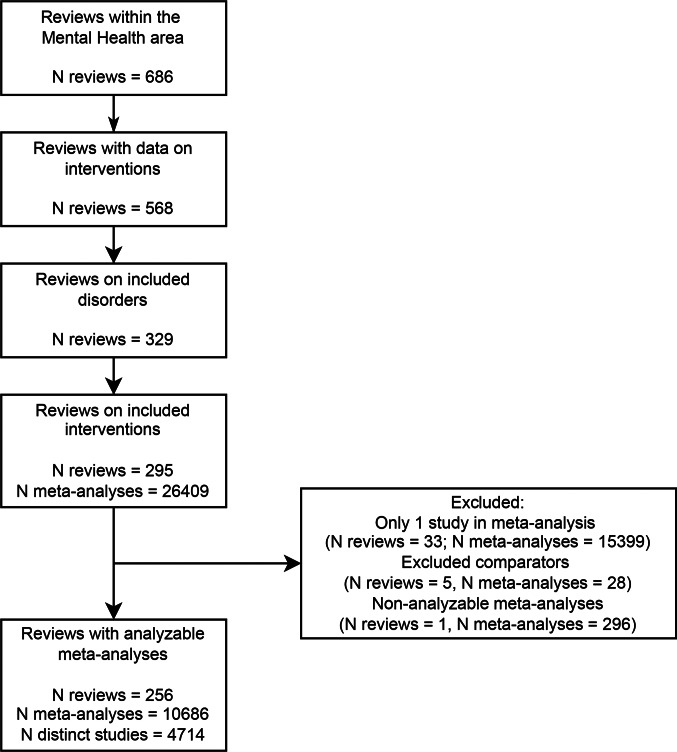
Flow chart of study selection process.

We received 686 reviews, of which 568 included usable data (see Fig. 1 for a flow chart). After exclusion of ineligible reviews (most commonly because the topic was dementia/cognition or a mixed group of mental disorders) and meta-analyses, we retained 256 reviews with 10 684 meta-analyses. Among these meta-analyses, 2843 concerned continuous efficacy outcomes, 295 continuous safety outcomes, 2296 binary efficacy outcomes, and 5250 binary safety outcomes. The final dataset contained 47 382 observations (i.e. studies), but many studies were included in multiple meta-analyses; there were only approximately 4714 distinct studies. Each review included on average 41.4 meta-analyses (median = 20.5, range = 1–436), while each meta-analysis included on average 4.3 studies (median = 3, range = 2–80).

### Effect sizes and power for continuous efficacy outcomes

**Fig. 2.**
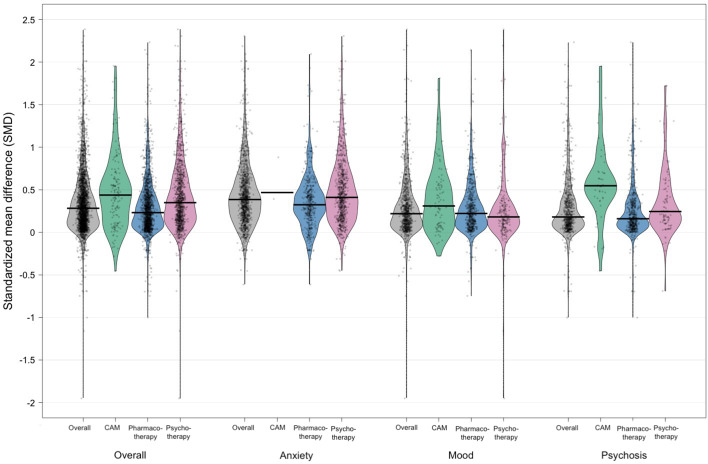
Distribution of meta-analytic effect sizes for continuous efficacy outcomes. Distributions are shown by disorder and intervention category. Dots indicate individual meta-analytic effect sizes, while the black bar represents the median meta-analytic effect size. The distribution is shown through a smoothed density.

Figure 2 shows the distribution of ES_MA_ for continuous efficacy outcomes (see also online Supplementary Table S1). The overall median effect size was 0.28 (interquartile range [IQR] = 0.11–0.52). Meta-analyses for anxiety disorders had larger effect sizes (median [IQR] = 0.39 [0.19–0.62]) than those for mood disorders (0.22 [0.09–0.41]) or psychotic disorders (0.18 [0.08–0.40]). Meta-analyses of CAM interventions also had larger effect sizes (0.44 [0.11–0.65]) than meta-analyses of PHT (0.23 [0.09–0.43]) or PST (0.35 [0.15–0.62]). These differences may be related, at least in part, to the comparators frequently used. Only 19% of meta-analyses for anxiety disorders compared the intervention with another similarly active comparator, compared to 36% of those for mood disorders and 65% of those for schizophrenia. Similarly, only 26% of CAM meta-analyses and 27% of PST meta-analyses compared the intervention with another similarly active comparator, compared to 43% of PHT meta-analyses. Effect sizes were larger for comparisons of active therapy with TAU/waitlist (median ES_MA_ = 0.48–0.70) or placebo/attention control (median ES_MA_ = 0.18–0.32), and of combination therapy with monotherapy (median ES_MA_ = 0.28–0.55), than for comparisons of monotherapy *v.* another monotherapy (median ES_MA_ = 0.14–27) (online Supplementary Table S2).

**Fig. 3.**
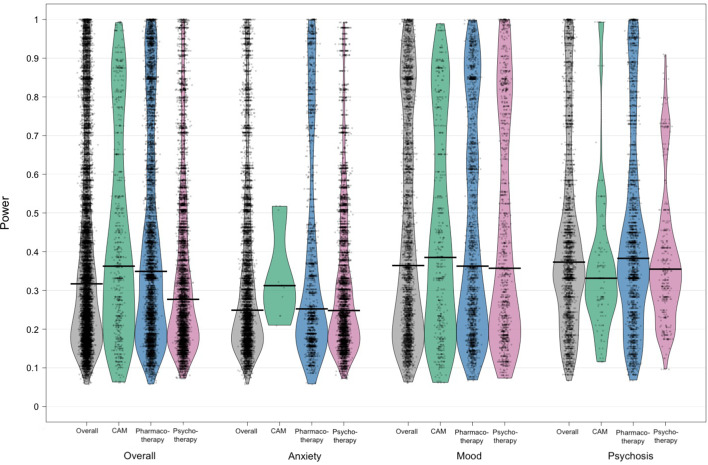
Distribution of power to detect SMD = 0.40 for continuous efficacy outcomes. Distributions are shown by disorder and intervention category. Dots indicate individual trial power estimates, while the black bar represents the median power estimate. The distribution is shown through a smoothed density.

Figure 3 shows the distribution of estimated power to detect SMD = 0.40 among studies with continuous efficacy outcomes (see also online Supplementary Table S3). Overall, median power was 0.32 (IQR = 0.19–0.54) and only 12.4% of studies were adequately-powered (⩾80%). Median power only exceeded the recommended threshold of 80% for SMD = 0.80 (0.84 [0.57–0.98]). Median power was slightly higher in studies of PHT (0.35 [0.20–0.64]) and CAM (0.36 [0.22–0.61]) than in studies of PST (0.28 [0.18–0.46]). It was also higher in studies of mood disorders (0.36 [0.20–0.73]) and psychotic disorders (0.37 [0.26–54]) than in studies of anxiety disorders (0.25 [0.17–0.43]). Consistent with the low median meta-analytic effect size (SMD = 0.28), power to detect the ES_MA_ was generally lower than the estimated power to detect an SMD = 0.40. Overall power to detect the ES_MA_ was only 0.23 [0.09–0.58] and 15.3% of studies were adequately-powered (⩾80%). Consistent with the differences in effect sizes, the power to detect the ES_MA_ was generally better in trials using TAU/waitlist (0.49–0.63) or placebo (0.12–0.38) as a comparator than in trials with active *v.* active comparisons (0.07–0.13) (see online Supplementary Table S4).

**Fig. 4.**
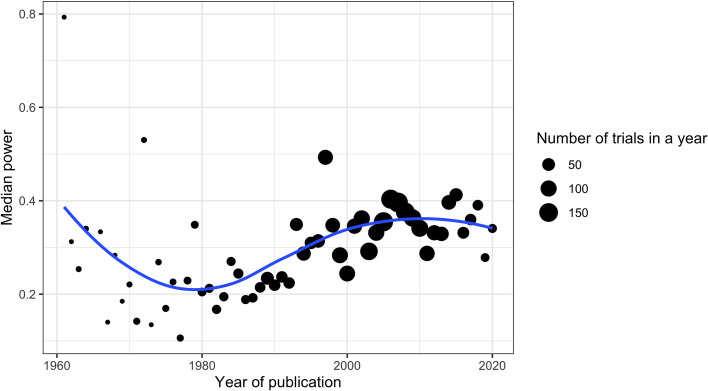
Median power by year of trial publication. Number of trials by year is indicated through the size of the dot. The line represents a Loess smoother.

Examining the trend in median power to detect an SMD = 0.40 over time suggested an increase in power, from a median of around 0.25 from 1960 until 1990, increasing to close to 0.40 in recent years, although this increase appears to have stalled recently (Fig. 4). This trend appeared to be present for each intervention type (online Supplementary Fig. S1).

### Effect sizes and power for other outcomes

Online Supplementary Tables S5 through S10 contain the median ES_MA_ by disorder and intervention type and by intervention-comparator combination for continuous safety outcomes and for binary safety and efficacy outcomes. Overall, the median ES_MA_ for continuous safety outcomes was SMD = 0.14 [IQR = 0.03–0.35]. The median ES_MA_ for binary efficacy and safety outcomes was OR = 1.39 [1.04–2.25] and OR = 1.32 [1.04–1.92], respectively. Online Supplementary Tables S11 through S16 provide detailed information on power to detect the full range of effect sizes by disorder and intervention type and by intervention-comparator combination for these outcomes. In brief, median power to detect SMD = 0.40 among trials examining a continuous safety outcome was quite high, at 0.78 [0.35–0.95]. However, median power to detect OR = 2.0 was 0.24 [0.16–0.44] for binary efficacy outcomes and 0.21 [0.13–0.39] for binary safety outcomes. Consistent with the low median ES_MA_ for all outcomes, power to detect the ES_MA_ was lower than the power to detect SMD = 0.40 or OR = 2.0. For binary efficacy outcomes, but not for safety outcomes, patterns mirrored those for continuous efficacy outcomes, with a higher power in trials using placebo or TAU/waitlist (0.46–0.54) than in trials with active *v.* active comparisons (0.07–0.23).

### Impact of underpowered studies on meta-analyses

For this analysis, 172 meta-analyses met inclusion criteria. On average, underpowered studies had an effect size (SMD) of 0.31, and there was a significant difference in effect size between adequately-powered and underpowered studies (*B* = −0.06, *p* < 0.001, *τ*^2^ = 0.01, *I*^2^ = 35%), indicating that adequately-powered studies had an effect size 0.06 (or about 20%) smaller than that of underpowered studies.

### Sensitivity analyses

We performed several sensitivity analyses for the continuous efficacy outcome (online Supplementary Tables S17–S19). Overall, the ES_avg_ was similar to the median ES_MA_ for most intervention-comparator combinations (online Supplementary Table S17), and power to detect the ES_avg_ was also similar to the power to detect the ES_MA_ found in our main analyses, but with less variation (0.20 [0.11–0.42] compared to 0.23 [0.09–0.58]). Exclusion of meta-analyses with very small effect sizes resulted in a small increase in overall median power to detect the ES_MA_ (to 0.43 [0.22–0.76]). Basing the ES_MA_ on the largest trial in a meta-analysis only slightly decreased the overall median power to detect the ES_MA_ (estimated at 0.17 [0.07–0.49]). Finally, estimates of power were nearly identical when we only included each study once (e.g. overall power to detect SMD = 0.40 was 0.33 [0.20–0.58] *v.* 0.32 [0.19–0.54] in our main analyses). This suggests that our main analyses were not overly influenced by a small subset of studies included in many meta-analyses.

## Discussion

### Principal findings

In this study, we provide a detailed examination of statistical power in psychiatric trials. As expected, we found that power is low: median power to detect a medium effect size (SMD = 0.40) was 0.32, well below recommended levels (80%). The median power to detect the meta-analysis-specific effect size (ES_MA_) was even lower, at only 0.23. Despite the fact that trials for different disorders and intervention types are performed by different teams of researchers, often working in somewhat siloed fields and in different contexts (e.g. academic *v.* industry), we found only small differences among the different disorders and different intervention types. However, trials that compared an active treatment to a less active treatment (e.g. pharmacotherapy *v.* placebo or psychotherapy compared to TAU/waitlist) had a much higher median power to detect the ES_MA_ (0.12–0.63) than trials that compared similarly active treatments (0.07–0.13).

We also examined binary efficacy outcomes as well as binary and continuous safety outcomes. Surprisingly, we found that the median power to detect SMD = 0.40 was relatively high for continuous safety outcomes (median power = 0.78). However, such outcomes (e.g. weight change) were uncommon and almost exclusively used in trials comparing two antipsychotics. As mental health trials are seldom powered specifically to detect safety issues, it seems more likely that these outcomes just happened to be included in large trials than that this was a deliberate attempt to adequately power these specific outcomes. In contrast, the median power to detect OR = 2.0 for binary outcomes was very low, at 0.21–0.24. These findings are fairly consistent with previous work by Turner et al. (2013), who found a median power of 0.14 for binary outcomes, and indicate that statistical power for the (usually) continuous primary outcome in psychiatric trials is actually somewhat better than suggested by previous work, although still inadequate. The lower power for binary outcomes compared to continuous outcomes reflects the fact that larger sample sizes are required to detect a similar effect size for binary outcomes. Given this, avoiding unnecessary dichotomization of continuous variables (e.g. into remission *v.* non-remission) is one way to increase statistical power.

### Implications and comparison with previous literature

It is generally recommended that trials should have a power of 80% to detect a desired effect size. This effect size might be the expected effect size based on previous literature [although this is fraught with difficulties (Anderson, Kelley, & Maxwell, 2017)] or the minimal clinically relevant effect size. Our findings suggest that trialists in the mental health field implicitly work under the assumption that SMD = 0.80 is a realistic or minimal clinically relevant effect size, as median power only exceeded the 80% threshold for this SMD. Realistically, however, effect sizes in psychiatry are commonly in the range of 0.20–0.60 (Huhn et al., 2014). The apparent tendency to expect very large effects may be, in part, a consequence of biases in the literature, which have led to inflated effect sizes. It may also be due to calculating power based on small pilot studies, which tend to overestimate effect size (if only statistically significant pilot studies are followed up) (Anderson et al., 2017). Effect sizes are not intuitive and commonly-used rules of thumb (e.g. that an SMD of 0.20 is ‘small’, 0.50 is ‘medium’, and 0.80 is ‘large’) may lead researchers to think that fairly large effect sizes are more likely than they are or that realistic (but small) effect sizes are clinically irrelevant. Lack of funding may also be a reason to limit sample size, particularly for non-industry-funded trials. On the other hand, trialists may have sometimes planned an adequate sample size but encountered problems in achieving this (e.g. due to difficulties in recruiting participants within a grant time frame, or higher than expected attrition); some of the included trials may also have had low power because they were intended as pilot studies. Additionally, outcome variability may have been greater than expected, reducing power. Future research could investigate the mechanisms behind our findings of low power across the mental health field.

We also found that active *v.* control comparisons had larger effect sizes than active *v.* active comparisons. This finding is probably unsurprising to almost everyone in the mental health field, so one might expect trialists to adjust their planned sample size accordingly and use larger samples in trials of active *v.* active comparisons. However, we find little indication that they do so at all, since the power to detect SMD = 0.40 is similar across comparators, implying that sample sizes in active *v.* active trials are similar to those in active *v.* control trials. As we are fortunate to have reached the point in psychiatry that several effective treatments are available for mood, anxiety and psychotic disorders, the question of real interest now is not ‘does this treatment work better than placebo/waitlist/care-as-usual?’ but ‘does this treatment work better than other treatments?’. Our findings imply that this question will be particularly difficult to answer with any confidence based on our current evidence base. These findings also demonstrate that previous findings suggesting much higher power for psychotherapy trials (Flint et al., 2015; Sakaluk, Williams, Kilshaw, & Rhyner, 2019) are largely due to the fact that psychotherapy is often compared to an inactive and problematic control condition [waitlist, which has previously been found to have a *nocebo* effect (Furukawa et al., 2014)]. Comparisons of psychotherapy to better control conditions with smaller effect sizes are just as underpowered as comparisons of other interventions.

Our results also show that statistical power is improving over time, although it remains well below recommended levels (80%). This is in contrast to previous work that found no increase in power over time (Lamberink et al., 2018; Smaldino & McElreath, 2016). This might suggest that trends are different in psychiatric clinical trials than in other areas. However, the difference may also be due to methodological differences, such as the fact that we specifically examined continuous efficacy outcomes and looked at power to detect an SMD of 0.40, rather than the ES_MA_. Unfortunately, the improvement in power over time also appears to have stalled out in the previous five years or so.

We also found that low power does have consequences for the published literature, as underpowered studies tended to yield higher effect sizes. This is consistent with previous work by Turner et al. (Turner et al., 2013), although the difference between underpowered and adequately-powered studies was somewhat larger in our study. This finding is consistent with reporting bias against underpowered studies with nonsignificant findings. It therefore remains important for meta-analysts to carefully consider the possible biasing effects of underpowered studies in a meta-analysis and to use methods to mitigate or explore these effects. However, the limited number of studies in most meta-analyses makes it difficult to address potential problems with underpowered studies.

### Strengths and limitations

An important strength of our study is that we used the highly comprehensive Cochrane dataset. Our analysis was also specific enough to illuminate possible differences among disorders, intervention types, comparators, and outcome types. Because trials are generally only powered to detect their primary outcome, our examination of continuous efficacy outcomes separately from safety and binary efficacy outcomes make the results more clearly applicable to clinicians. We also examined power from multiple angles, including the power to detect both predetermined and meta-analytic effect sizes. The fine-grained nature of our analysis adds important new information to previous studies, for instance regarding the differences among comparators.

Our study also has several limitations. Some of these limitations may have led to an overestimate of power due to an overestimate of effect sizes. First, since our analysis was based on the published literature, estimated effect sizes may be inflated due to reporting bias. Second, we used the absolute effect size for comparisons of two active treatments, as the direction of effects is somewhat arbitrary. This may have led to an overestimate of ES_avg_ (although not ES_MA_). These limitations imply that the problem of low power may actually be even greater than our results already suggest. On the other hand, similar to previous studies, we did not determine the primary outcome of each of the nearly 5000 included trials. Therefore, it is likely that we also included secondary outcomes for which trials were not explicitly powered, given that we included, on average, about four continuous efficacy outcomes per study. Secondary outcomes may have systematically smaller effect sizes, as trialists presumably often select the outcome they expect to be most directly affected by an intervention as the primary outcome. However, all of these limitations would only affect our analyses based on the ES_MA_ and/or ES_avg_ and not our main analyses based on a predetermined effect size (SMD = 0.40), as these are only dependent on sample size and outcome type.

### Conclusions

In this examination of the comprehensive Cochrane database, we found that power was somewhat better than might have been expected based on previous research, but still highly inadequate. Median power has increased somewhat over time, but remains far below the recommended 80% level. Power was low regardless of the specific disorder or intervention under investigation. Our findings suggest that trialists are implicitly working under the assumption that very large effect sizes are realistic and do not adjust sample sizes for different types of trials, in particular for trials with more *v.* less active comparators. Consequently, head-to-head trials are especially likely to be underpowered to detect realistic effect sizes, which may pose a significant obstacle to the project of precision medicine. Importantly, underpowered studies resulted in higher effect sizes than adequately powered studies, consistent with the presence of reporting bias. These findings emphasize the urgent need to increase sample sizes in clinical trials and to reduce reporting bias against studies with nonsignificant results to improve the reliability of the published literature.

## Supplementary material

For supplementary material accompanying this paper visit https://doi.org/10.1017/S0033291722001362. click here to view supplementary material
