# Supplementary material for: Statistical power in clinical trials of interventions for mood, anxiety, and psychotic disorders
Source: Psychol Med. 2022 May 19;53(10):4499–506. doi: 10.1017/S0033291722001362 (PMC10388329; doi:10.1017/S0033291722001362)
Supplement: Supplementary file 1 [file S0033291722001362sup.zip › S0033291722001362sup002.docx]

**Supplemental table 1. Median meta-analysis effect size by disorder and intervention category for continuous efficacy outcomes.**

| **Disorder** | **Intervention** | **N** | | | **Meta-analysis SMD** |
| --- | --- | --- | --- | --- | --- |
|  |  | **Meta-analyses** | **Studies** | **Unique studies** | **Median (IQR)** |
| **Overall** | **Overall** | 2843 | 13653 | 2841 | 0.28 (0.11 - 0.52) |
|  | **CAM** | 141 | 637 | 216 | 0.44 (0.11 - 0.65) |
|  | **Pharmacotherapy** | 1604 | 6554 | 1750 | 0.23 (0.09 - 0.43) |
|  | **Psychotherapy** | 1098 | 6462 | 886 | 0.35 (0.15 - 0.62) |
| **Anxiety** | **Overall** | 1215 | 6839 | 824 | 0.39 (0.19 - 0.62) |
|  | **CAM** | 3 | 11 | 9 | 0.47 (0.43 - 0.67) |
|  | **Pharmacotherapy** | 439 | 1889 | 283 | 0.32 (0.17 - 0.50) |
|  | **Psychotherapy** | 773 | 4939 | 541 | 0.41 (0.20 - 0.68) |
| **Mood** | **Overall** | 893 | 3943 | 1166 | 0.22 (0.09 - 0.41) |
|  | **CAM** | 92 | 502 | 147 | 0.31 (0.08 - 0.58) |
|  | **Pharmacotherapy** | 581 | 2301 | 755 | 0.22 (0.09 - 0.41) |
|  | **Psychotherapy** | 220 | 1140 | 266 | 0.18 (0.06 - 0.35) |
| **Psychosis** | **Overall** | 735 | 2871 | 864 | 0.18 (0.08 - 0.40) |
|  | **CAM** | 46 | 124 | 60 | 0.55 (0.40 - 0.71) |
|  | **Pharmacotherapy** | 584 | 2364 | 714 | 0.16 (0.07 - 0.35) |
|  | **Psychotherapy** | 105 | 383 | 90 | 0.24 (0.12 - 0.51) |

*Table notes: For active vs. active comparisons, absolute effect sizes were used, as the experimental and comparator conditions are essentially interchangeable. CAM = Complementary and alternative medicine. SMD = standardized mean difference.*

**Supplemental table 2: Median meta-analysis effect size by experimental group and comparator for continuous efficacy outcomes.**

| **Experimental group** | **Comparator** | **N** | | | **Meta-analysis SMD** |
| --- | --- | --- | --- | --- | --- |
|  |  | **Meta-analyses** | **Studies** | **Unique studies** | **Median (IQR)** |
| Pharmacotherapy (mono) | Placebo | 611 | 2544 | 588 | 0.32 (0.20 - 0.53) |
|  | Pharmacotherapy (mono) | 689 | 2813 | 1023 | 0.14 (0.07 - 0.27) |
| Combination pharmacotherapy | Pharmacotherapy (mono) | 120 | 457 | 138 | 0.33 (0.11 - 0.62) |
| Pharmacotherapy + psychotherapy | Psychotherapy | 181 | 731 | 54 | 0.28 (0.09 - 0.49) |
|  | Pharmacotherapy (mono) | 87 | 216 | 25 | 0.36 (0.19 - 0.55) |
| Psychotherapy | Placebo/attention control | 29 | 167 | 72 | 0.18 (0.04 - 0.35) |
|  | Treatment-as-usual/waitlist | 588 | 4188 | 622 | 0.48 (0.21 - 0.80) |
|  | Psychotherapy | 279 | 1343 | 199 | 0.24 (0.13 - 0.38) |
|  | Pharmacotherapy | 16 | 64 | 21 | 0.15 (0.10 - 0.18) |
|  | Any comparator | 96 | 477 | 56 | 0.24 (0.08 - 0.40) |
| CAM + regular treatment | Pharmacotherapy (mono) | 31 | 83 | 45 | 0.55 (0.40 - 0.85) |
| CAM | Placebo | 46 | 320 | 90 | 0.30 (0.06 - 0.53) |
|  | Treatment-as-usual/waitlist | 31 | 89 | 51 | 0.70 (0.43 - 1.00) |
|  | Any active comparator | 39 | 161 | 82 | 0.27 (0.08 - 0.47) |

*Table notes: CAM = complementary and alternative medicine. SMD = standardized mean difference.*

**Supplemental table 3: Median power for a range of effect sizes and the ES_MA_ by disorder and intervention category, for continuous efficacy outcomes**

| **Disorder** | **Intervention** | **Power** | | | | |
| --- | --- | --- | --- | --- | --- | --- |
|  |  | **SMD=0.20** | **SMD=0.40** | **SMD=0.60** | **SMD=0.80** | **ES_MA_** |
|  |  | **Median (IQR)** | **Median (IQR)** | **Median (IQR)** | **Median (IQR)** | **Median (IQR)** |
| **Overall** | **Overall** | 0.12 (0.08 - 0.18) | 0.32 (0.19 - 0.54) | 0.60 (0.36 - 0.87) | 0.84 (0.57 - 0.98) | 0.23 (0.09 - 0.58) |
|  | **CAM** | 0.13 (0.09 - 0.20) | 0.36 (0.22 - 0.61) | 0.67 (0.43 - 0.92) | 0.90 (0.66 - 0.99) | 0.29 (0.10 - 0.77) |
|  | **Pharmacotherapy** | 0.12 (0.09 - 0.21) | 0.35 (0.20 - 0.64) | 0.65 (0.40 - 0.94) | 0.88 (0.62 - 1.00) | 0.16 (0.07 - 0.44) |
|  | **Psychotherapy** | 0.10 (0.08 - 0.15) | 0.28 (0.18 - 0.46) | 0.54 (0.35 - 0.79) | 0.78 (0.56 - 0.96) | 0.33 (0.12 - 0.68) |
| **Anxiety** | **Overall** | 0.10 (0.08 - 0.15) | 0.25 (0.17 - 0.43) | 0.48 (0.33 - 0.77) | 0.73 (0.53 - 0.95) | 0.36 (0.14 - 0.70) |
|  | **CAM** | 0.11 (0.10 - 0.14) | 0.31 (0.26 - 0.41) | 0.60 (0.50 - 0.73) | 0.84 (0.74 - 0.91) | 0.31 (0.25 - 0.57) |
|  | **Pharmacotherapy** | 0.10 (0.08 - 0.19) | 0.25 (0.17 - 0.59) | 0.49 (0.33 - 0.91) | 0.73 (0.53 - 0.99) | 0.24 (0.10 - 0.56) |
|  | **Psychotherapy** | 0.10 (0.08 - 0.14) | 0.25 (0.17 - 0.42) | 0.48 (0.33 - 0.75) | 0.72 (0.52 - 0.94) | 0.40 (0.17 - 0.74) |
| **Mood** | **Overall** | 0.13 (0.09 - 0.25) | 0.36 (0.20 - 0.73) | 0.68 (0.40 - 0.97) | 0.90 (0.62 - 1.00) | 0.18 (0.08 - 0.45) |
|  | **CAM** | 0.13 (0.09 - 0.24) | 0.39 (0.21 - 0.71) | 0.71 (0.41 - 0.96) | 0.92 (0.63 - 1.00) | 0.22 (0.09 - 0.72) |
|  | **Pharmacotherapy** | 0.13 (0.09 - 0.24) | 0.36 (0.21 - 0.72) | 0.67 (0.41 - 0.97) | 0.90 (0.63 - 1.00) | 0.19 (0.08 - 0.45) |
|  | **Psychotherapy** | 0.13 (0.09 - 0.26) | 0.36 (0.19 - 0.76) | 0.67 (0.38 - 0.98) | 0.89 (0.59 - 1.00) | 0.15 (0.07 - 0.37) |
| **Psychosis** | **Overall** | 0.13 (0.10 - 0.18) | 0.37 (0.26 - 0.54) | 0.69 (0.51 - 0.87) | 0.91 (0.76 - 0.98) | 0.10 (0.06 - 0.33) |
|  | **CAM** | 0.12 (0.10 - 0.14) | 0.33 (0.26 - 0.41) | 0.63 (0.51 - 0.74) | 0.86 (0.76 - 0.93) | 0.59 (0.33 - 0.96) |
|  | **Pharmacotherapy** | 0.13 (0.10 - 0.19) | 0.38 (0.26 - 0.58) | 0.70 (0.51 - 0.90) | 0.91 (0.76 - 0.99) | 0.09 (0.06 - 0.26) |
|  | **Psychotherapy** | 0.12 (0.10 - 0.15) | 0.36 (0.28 - 0.44) | 0.66 (0.53 - 0.78) | 0.89 (0.78 - 0.95) | 0.15 (0.08 - 0.37) |

*Table notes: CAM = complementary and alternative medicine. SMD = standardized mean difference. ES_MA_ = meta-analytic effect size.*

**Supplemental table 4: Power to detect SMD=0.40 and the ES_MA_ for each combination of experimental group and comparator for continuous efficacy outcomes.**

| **Experimental group** | **Comparator** | **Power** | |
| --- | --- | --- | --- |
|  |  | **SMD=0.40** | **ES_MA_** |
|  |  | **Median (IQR)** | **Median (IQR)** |
| Pharmacotherapy (mono) | Placebo | 0.39 (0.20 - 0.80) | 0.38 (0.15 - 0.75) |
|  | Pharmacotherapy (mono) | 0.41 (0.28 - 0.64) | 0.08 (0.06 - 0.17) |
| Combination pharmacotherapy | Pharmacotherapy (mono) | 0.20 (0.14 - 0.33) | 0.19 (0.08 - 0.45) |
| Pharmacotherapy + psychotherapy | Psychotherapy | 0.19 (0.17 - 0.26) | 0.16 (0.08 - 0.30) |
|  | Pharmacotherapy (mono) | 0.23 (0.17 - 0.32) | 0.20 (0.09 - 0.39) |
| Psychotherapy | Placebo/attention control | 0.24 (0.18 - 0.37) | 0.12 (0.07 - 0.26) |
|  | Treatment-as-usual/waitlist | 0.28 (0.18 - 0.46) | 0.49 (0.24 - 0.81) |
|  | Psychotherapy | 0.26 (0.17 - 0.42) | 0.12 (0.07 - 0.22) |
|  | Pharmacotherapy | 0.22 (0.19 - 0.33) | 0.07 (0.06 - 0.10) |
|  | Any comparator | 0.35 (0.19 - 0.62) | 0.23 (0.10 - 0.57) |
| CAM + regular treatment | Pharmacotherapy (mono) | 0.33 (0.23 - 0.47) | 0.62 (0.34 - 0.99) |
| CAM | Placebo | 0.42 (0.23 - 0.71) | 0.25 (0.10 - 0.76) |
|  | TAU/waitlist | 0.28 (0.20 - 0.36) | 0.63 (0.30 - 0.96) |
|  | Any active comparator | 0.37 (0.21 - 0.77) | 0.13 (0.07 - 0.34) |

*Table notes: CAM = complementary and alternative medicine. SMD = standardized mean difference. ES_MA_ = meta-analytic effect size.*

**Supplemental table 5: Median meta-analysis effect size by disorder and intervention category for continuous safety outcomes.**

| **Disorder** | **Intervention** | **N** | | | **Meta-analysis SMD** |
| --- | --- | --- | --- | --- | --- |
|  |  | **Meta-analyses** | **Studies** | **Unique studies** | **Median (IQR)** |
| **Overall** | **Overall** | 295 | 911 | 279 | 0.14 (0.03 - 0.35) |
|  | **CAM** | 4 | 9 | 9 | 0.46 (0.21 - 0.89) |
|  | **Pharmacotherapy** | 284 | 888 | 265 | 0.13 (0.03 - 0.35) |
|  | **Psychotherapy** | 7 | 14 | 7 | 0.20 (0.03 - 0.27) |
| **Anxiety** | **Overall** | 8 | 25 | 16 | 0.16 (0.03 - 0.61) |
|  | **CAM** | 0 | 0 | 0 | - |
|  | **Pharmacotherapy** | 7 | 23 | 14 | 0.16 (0.12 - 0.70) |
|  | **Psychotherapy** | 1 | 2 | 2 | -0.10 (-0.10 - -0.10) |
| **Mood** | **Overall** | 34 | 76 | 47 | -0.04 (-0.28 - 0.28) |
|  | **CAM** | 2 | 5 | 5 | 1.14 (0.75 - 1.52) |
|  | **Pharmacotherapy** | 27 | 61 | 41 | -0.07 (-0.38 - 0.16) |
|  | **Psychotherapy** | 5 | 10 | 3 | 0.27 (0.20 - 0.28) |
| **Psychosis** | **Overall** | 253 | 810 | 216 | 0.16 (0.05 - 0.35) |
|  | **CAM** | 2 | 4 | 4 | 0.14 (-0.06 - 0.35) |
|  | **Pharmacotherapy** | 250 | 804 | 210 | 0.16 (0.05 - 0.35) |
|  | **Psychotherapy** | 1 | 2 | 2 | -0.11 (-0.11 - -0.11) |

*Table notes: For active vs. active comparisons, absolute effect sizes were used, as the experimental and comparator conditions are essentially interchangeable. CAM = complementary and alternative medicine. SMD = standardized mean difference.*

**Supplemental table 6: Median meta-analysis effect size by disorder and intervention category for binary efficacy outcomes.**

| **Disorder** | **Intervention** | **N** | | | **Meta-analysis OR** |
| --- | --- | --- | --- | --- | --- |
|  |  | **Meta-analyses** | **Studies** | **Unique studies** | **Median (IQR)** |
| **Overall** | **Overall** | 2296 | 11236 | 2734 | 1.39 (1.04 - 2.25) |
|  | **CAM** | 66 | 303 | 128 | 1.22 (0.65 - 2.43) |
|  | **Pharmacotherapy** | 1772 | 8471 | 2131 | 1.34 (1.03 - 2.14) |
|  | **Psychotherapy** | 458 | 2462 | 486 | 1.71 (1.11 - 2.77) |
| **Anxiety** | **Overall** | 536 | 2931 | 487 | 1.16 (0.54 - 2.31) |
|  | **CAM** | 1 | 2 | 2 | 12.37 (12.37 - 12.37) |
|  | **Pharmacotherapy** | 310 | 1583 | 257 | 0.72 (0.44 - 1.58) |
|  | **Psychotherapy** | 225 | 1346 | 236 | 1.89 (1.07 - 3.93) |
| **Mood** | **Overall** | 1038 | 4391 | 1081 | 1.34 (1.07 - 1.99) |
|  | **CAM** | 47 | 260 | 97 | 1.09 (0.51 - 1.39) |
|  | **Pharmacotherapy** | 876 | 3545 | 866 | 1.34 (1.09 - 2.00) |
|  | **Psychotherapy** | 115 | 586 | 121 | 1.44 (1.04 - 1.97) |
| **Psychosis** | **Overall** | 722 | 3914 | 1166 | 1.68 (1.16 - 2.81) |
|  | **CAM** | 18 | 41 | 29 | 2.63 (1.65 - 6.20) |
|  | **Pharmacotherapy** | 586 | 3343 | 1008 | 1.58 (1.13 - 2.90) |
|  | **Psychotherapy** | 118 | 530 | 129 | 1.85 (1.25 - 2.38) |

*Table notes: For active vs. active comparisons, we used the inverse for ORs > 1, as the experimental and comparator conditions are essentially interchangeable. CAM = Complementary and alternative medicine. OR = odds ratio.*

**Supplemental table 7: Median meta-analysis effect size by disorder and intervention category for binary safety outcomes.**

| **Disorder** | **Intervention** | **N** | | | **Meta-analysis OR** |
| --- | --- | --- | --- | --- | --- |
|  |  | **Meta-analyses** | **Studies** | **Unique studies** | **Median (IQR)** |
| **Overall** | **Overall** | 5250 | 21582 | 3813 | 1.32 (1.04 - 1.92) |
|  | **CAM** | 127 | 534 | 212 | 1.28 (1.07 - 2.12) |
|  | **Pharmacotherapy** | 4859 | 19537 | 2990 | 1.34 (1.05 - 1.96) |
|  | **Psychotherapy** | 264 | 1511 | 621 | 1.12 (0.84 - 1.49) |
| **Anxiety** | **Overall** | 470 | 2327 | 660 | 0.94 (0.57 - 1.45) |
|  | **CAM** | 4 | 8 | 4 | 1.52 (1.15 - 1.94) |
|  | **Pharmacotherapy** | 337 | 1587 | 311 | 0.85 (0.47 - 1.36) |
|  | **Psychotherapy** | 129 | 732 | 352 | 1.05 (0.74 - 1.47) |
| **Mood** | **Overall** | 2070 | 7743 | 1319 | 1.30 (1.05 - 1.79) |
|  | **CAM** | 47 | 286 | 100 | 1.26 (0.99 - 1.61) |
|  | **Pharmacotherapy** | 1943 | 6998 | 1112 | 1.31 (1.06 - 1.80) |
|  | **Psychotherapy** | 80 | 459 | 110 | 1.12 (0.93 - 1.38) |
| **Psychosis** | **Overall** | 2710 | 11512 | 1834 | 1.40 (1.09 - 2.11) |
|  | **CAM** | 76 | 240 | 108 | 1.36 (1.08 - 2.74) |
|  | **Pharmacotherapy** | 2579 | 10952 | 1567 | 1.41 (1.09 - 2.10) |
|  | **Psychotherapy** | 55 | 320 | 159 | 1.20 (1.06 - 1.87) |

*Table notes: For active vs. active comparisons, we used the inverse for ORs > 1, as the experimental and comparator conditions are essentially interchangeable. CAM = complementary and alternative medicine. OR = odds ratio.*

**Supplemental table 8: Median meta-analysis effect size by experimental group and comparator for continuous safety outcomes.**

| **Experimental group** | **Comparator** | **N** | | | **Meta-analysis SMD** |
| --- | --- | --- | --- | --- | --- |
|  |  | **Meta-analyses** | **Studies** | **Unique studies** | **Median (IQR)** |
| Pharmacotherapy (mono) | Placebo | 54 | 184 | 58 | -0.06 (-0.28 - 0.03) |
|  | Pharmacotherapy (mono) | 223 | 687 | 200 | 0.20 (0.08 - 0.41) |
| Combination pharmacotherapy | Pharmacotherapy (mono) | 6 | 15 | 13 | -0.38 (-1.32 - -0.16) |
| Psychotherapy | Any comparison | 7 | 14 | 7 | 0.20 (0.03 - 0.27) |
| CAM | Any comparison | 5 | 11 | 11 | 0.56 (0.37 - 0.86) |

*Table notes: CAM = complementary and alternative medicine. SMD = standardized mean difference.*

**Supplemental table 9: Median meta-analysis effect size by experimental group and comparator for binary efficacy outcomes.**

| **Experimental group** | **Comparator** | **N** | | | **Meta-analysis SMD** |
| --- | --- | --- | --- | --- | --- |
|  |  | **Meta-analyses** | **Studies** | **Unique studies** | **Median (IQR)** |
| Pharmacotherapy (mono) | Placebo | 784 | 4351 | 766 | 1.77 (0.67 - 3.32) |
|  | Pharmacotherapy (mono) | 778 | 3110 | 1263 | 1.29 (1.12 - 1.60) |
| Combination pharmacotherapy | Pharmacotherapy (mono) | 105 | 563 | 124 | 1.56 (0.63 - 2.27) |
| Pharmacotherapy + psychotherapy | Psychotherapy | 104 | 445 | 42 | 0.67 (0.60 - 0.96) |
|  | Pharmacotherapy (mono) | 50 | 126 | 16 | 0.57 (0.42 - 1.01) |
| Psychotherapy | Placebo/attention control | 15 | 83 | 16 | 3.06 (1.58 - 3.88) |
|  | Treatment-as-usual/waitlist | 195 | 1364 | 326 | 2.27 (1.42 - 4.87) |
|  | Psychotherapy | 118 | 518 | 118 | 1.52 (1.14 - 2.01) |
|  | Pharmacotherapy | 46 | 242 | 22 | 1.58 (1.22 - 1.89) |
|  | Any comparator | 31 | 121 | 19 | 1.71 (0.41 - 2.18) |
| CAM + regular treatment | Any active comparator | 15 | 40 | 29 | 2.49 (1.40 - 4.52) |
| CAM | Placebo/treatment-as-usual/waitlist | 30 | 147 | 61 | 0.60 (0.39 - 1.18) |
|  | Any active comparator | 25 | 126 | 57 | 1.24 (1.13 - 1.95) |

*Table notes: CAM = complementary and alternative medicine. OR = odds ratio.*

**Supplemental table 10: Median meta-analysis effect size by experimental group and comparator for binary safety outcomes.**

| **Experimental group** | **Comparator** | **N** | | | **Meta-analysis OR** |
| --- | --- | --- | --- | --- | --- |
|  |  | **Meta-analyses** | **Studies** | **Unique studies** | **Median (IQR)** |
| Pharmacotherapy (mono) | Placebo | 1222 | 5319 | 919 | 0.80 (0.46 - 1.26) |
|  | Pharmacotherapy (mono) | 3406 | 13170 | 1942 | 1.50 (1.20 - 2.21) |
| Combination pharmacotherapy | Pharmacotherapy (mono) | 165 | 765 | 196 | 0.83 (0.49 - 1.35) |
| Pharmacotherapy + psychotherapy | Psychotherapy | 65 | 278 | 49 | 0.78 (0.32 - 0.92) |
|  | Pharmacotherapy (mono) | 34 | 90 | 22 | 1.17 (0.89 - 1.51) |
| Psychotherapy | Placebo/attention control | 6 | 32 | 25 | 0.95 (0.85 - 1.14) |
|  | Treatment-as-usual/waitlist | 112 | 702 | 385 | 0.95 (0.65 - 1.32) |
|  | Psychotherapy | 74 | 435 | 180 | 1.23 (1.09 - 1.88) |
|  | Pharmacotherapy | 13 | 53 | 20 | 1.65 (1.18 - 2.27) |
|  | Any comparator | 24 | 197 | 54 | 0.79 (0.64 - 1.15) |
| CAM + regular treatment | Pharmacotherapy (mono) | 50 | 152 | 53 | 1.65 (1.14 - 2.85) |
| CAM | Placebo | 31 | 206 | 75 | 1.01 (0.69 - 1.17) |
|  | Treatment-as-usual/waitlist | 12 | 58 | 51 | 1.20 (0.93 - 1.61) |
|  | Any active comparator | 36 | 125 | 61 | 1.61 (1.29 - 3.06) |

*Table notes: CAM = complementary and alternative medicine. OR = odds ratio.*

**Supplemental table 11: Median power for a range of effect sizes and the ES_MA_ by disorder and intervention category, for continuous safety outcomes**

| **Disorder** | **Inter-vention** | **Power** | | | | |
| --- | --- | --- | --- | --- | --- | --- |
|  |  | **SMD=0.20** | **SMD=0.40** | **SMD=0.60** | **SMD=0.80** | **ES_MA_** |
|  |  | **Median (IQR)** | **Median (IQR)** | **Median (IQR)** | **Median (IQR)** | **Median (IQR)** |
| **Overall** | **Overall** | 0.27 (0.12 - 0.43) | 0.78 (0.35 - 0.95) | 0.98 (0.66 - 1.00) | 1.00 (0.88 - 1.00) | 0.20 (0.08 - 0.76) |
|  | **CAM** | 0.13 (0.12 - 0.16) | 0.37 (0.33 - 0.47) | 0.68 (0.63 - 0.80) | 0.90 (0.86 - 0.96) | 0.41 (0.29 - 1.00) |
|  | **Pharmacotherapy** | 0.28 (0.13 - 0.44) | 0.78 (0.37 - 0.95) | 0.98 (0.69 - 1.00) | 1.00 (0.91 - 1.00) | 0.20 (0.08 - 0.77) |
|  | **Psychotherapy** | 0.15 (0.11 - 0.31) | 0.43 (0.28 - 0.83) | 0.75 (0.55 - 0.99) | 0.92 (0.79 - 1.00) | 0.16 (0.09 - 0.37) |
| **Anxiety** | **Overall** | 0.35 (0.23 - 0.52) | 0.89 (0.69 - 0.98) | 1.00 (0.96 - 1.00) | 1.00 (1.00 - 1.00) | 0.28 (0.17 - 1.00) |
|  | **CAM** | - | - | - | - | - |
|  | **Pharmacotherapy** | 0.35 (0.25 - 0.52) | 0.89 (0.72 - 0.98) | 1.00 (0.97 - 1.00) | 1.00 (1.00 - 1.00) | 0.93 (0.20 - 1.00) |
|  | **Psychotherapy** | 0.27 (0.17 - 0.37) | 0.56 (0.36 - 0.76) | 0.65 (0.47 - 0.82) | 0.74 (0.61 - 0.87) | 0.11 (0.08 - 0.13) |
| **Mood** | **Overall** | 0.26 (0.16 - 0.38) | 0.74 (0.48 - 0.91) | 0.98 (0.82 - 1.00) | 1.00 (0.97 - 1.00) | 0.46 (0.10 - 0.93) |
|  | **CAM** | 0.13 (0.12 - 0.16) | 0.37 (0.33 - 0.47) | 0.68 (0.63 - 0.80) | 0.90 (0.86 - 0.96) | 1.00 (0.41 - 1.00) |
|  | **Pharmacotherapy** | 0.28 (0.18 - 0.39) | 0.79 (0.55 - 0.92) | 0.98 (0.88 - 1.00) | 1.00 (0.99 - 1.00) | 0.62 (0.08 - 0.95) |
|  | **Psychotherapy** | 0.18 (0.11 - 0.31) | 0.55 (0.29 - 0.83) | 0.88 (0.56 - 0.99) | 0.99 (0.80 - 1.00) | 0.27 (0.15 - 0.41) |
| **Psychosis** | **Overall** | 0.27 (0.12 - 0.45) | 0.77 (0.33 - 0.95) | 0.98 (0.63 - 1.00) | 1.00 (0.86 - 1.00) | 0.18 (0.08 - 0.71) |
|  | **CAM** | 0.12 (0.09 - 0.17) | 0.33 (0.23 - 0.49) | 0.61 (0.46 - 0.81) | 0.82 (0.69 - 0.96) | 0.30 (0.20 - 0.52) |
|  | **Pharmacotherapy** | 0.27 (0.12 - 0.45) | 0.78 (0.34 - 0.95) | 0.98 (0.64 - 1.00) | 1.00 (0.87 - 1.00) | 0.18 (0.08 - 0.71) |
|  | **Psychotherapy** | 0.11 (0.11 - 0.11) | 0.31 (0.29 - 0.32) | 0.58 (0.57 - 0.60) | 0.82 (0.81 - 0.84) | 0.07 (0.07 - 0.07) |

*Table notes: CAM = complementary and alternative medicine. SMD = standardized mean difference. ES_MA_ = meta-analytic effect size.*

**Supplemental table 12: Median power for a range of effect sizes and the ES_MA_ by disorder and intervention category, for binary efficacy outcomes**

| **Disorder** | **Inter-vention** | **Power** | | | | |
| --- | --- | --- | --- | --- | --- | --- |
|  |  | **OR=1.5** | **OR=2.0** | **OR=3.0** | **OR=4.5** | **ES_MA_** |
|  |  | **Median (IQR)** | **Median (IQR)** | **Median (IQR)** | **Median (IQR)** | **Median (IQR)** |
| **Overall** | **Overall** | 0.11 (0.09 - 0.19) | 0.24 (0.16 - 0.44) | 0.50 (0.32 - 0.82) | 0.75 (0.51 - 0.97) | 0.23 (0.08 - 0.58) |
|  | **CAM** | 0.16 (0.10 - 0.27) | 0.36 (0.20 - 0.62) | 0.70 (0.42 - 0.94) | 0.91 (0.66 - 1.00) | 0.15 (0.06 - 0.57) |
|  | **Pharmacotherapy** | 0.12 (0.09 - 0.22) | 0.25 (0.16 - 0.53) | 0.53 (0.32 - 0.89) | 0.79 (0.53 - 0.99) | 0.21 (0.07 - 0.55) |
|  | **Psychotherapy** | 0.10 (0.08 - 0.13) | 0.21 (0.15 - 0.30) | 0.43 (0.29 - 0.60) | 0.66 (0.47 - 0.85) | 0.30 (0.10 - 0.67) |
| **Anxiety** | **Overall** | 0.11 (0.09 - 0.16) | 0.23 (0.16 - 0.37) | 0.46 (0.32 - 0.72) | 0.69 (0.50 - 0.92) | 0.42 (0.15 - 0.77) |
|  | **CAM** | 0.06 (0.06 - 0.06) | 0.07 (0.07 - 0.07) | 0.10 (0.10 - 0.10) | 0.13 (0.13 - 0.14) | 0.20 (0.20 - 0.21) |
|  | **Pharmacotherapy** | 0.14 (0.10 - 0.26) | 0.31 (0.18 - 0.61) | 0.61 (0.36 - 0.93) | 0.84 (0.56 - 1.00) | 0.33 (0.13 - 0.74) |
|  | **Psychotherapy** | 0.10 (0.08 - 0.13) | 0.20 (0.15 - 0.28) | 0.41 (0.29 - 0.57) | 0.63 (0.45 - 0.81) | 0.50 (0.18 - 0.78) |
| **Mood** | **Overall** | 0.13 (0.09 - 0.25) | 0.28 (0.16 - 0.59) | 0.57 (0.34 - 0.93) | 0.82 (0.54 - 1.00) | 0.17 (0.07 - 0.41) |
|  | **CAM** | 0.17 (0.10 - 0.30) | 0.39 (0.21 - 0.67) | 0.74 (0.44 - 0.96) | 0.93 (0.68 - 1.00) | 0.13 (0.06 - 0.53) |
|  | **Pharmacotherapy** | 0.13 (0.09 - 0.26) | 0.29 (0.17 - 0.62) | 0.60 (0.35 - 0.94) | 0.84 (0.56 - 1.00) | 0.19 (0.08 - 0.41) |
|  | **Psychotherapy** | 0.10 (0.08 - 0.15) | 0.20 (0.14 - 0.33) | 0.40 (0.25 - 0.67) | 0.62 (0.40 - 0.90) | 0.11 (0.06 - 0.30) |
| **Psychosis** | **Overall** | 0.11 (0.08 - 0.16) | 0.22 (0.15 - 0.37) | 0.47 (0.31 - 0.74) | 0.72 (0.51 - 0.95) | 0.19 (0.06 - 0.58) |
|  | **CAM** | 0.11 (0.08 - 0.13) | 0.23 (0.15 - 0.31) | 0.52 (0.33 - 0.65) | 0.81 (0.54 - 0.91) | 0.49 (0.10 - 0.74) |
|  | **Pharmacotherapy** | 0.10 (0.08 - 0.16) | 0.21 (0.15 - 0.39) | 0.46 (0.29 - 0.76) | 0.72 (0.49 - 0.95) | 0.19 (0.06 - 0.62) |
|  | **Psychotherapy** | 0.12 (0.09 - 0.15) | 0.25 (0.19 - 0.35) | 0.53 (0.39 - 0.72) | 0.80 (0.64 - 0.93) | 0.18 (0.09 - 0.38) |

*Table notes: CAM = complementary and alternative medicine. OR = odds ratio. ES_MA_ = meta-analytic effect size*

**Supplemental table 13: Median power for a range of effect sizes and the ES_MA_ by disorder and intervention category, for binary safety outcomes**

| **Disorder** | **Inter-vention** | **Power** | | | | |
| --- | --- | --- | --- | --- | --- | --- |
|  |  | **OR=1.5** | **OR=2.0** | **OR=3.0** | **OR=4.5** | **ES_MA_** |
|  |  | **Median (IQR)** | **Median (IQR)** | **Median (IQR)** | **Median (IQR)** | **Median (IQR)** |
| **Overall** | **Overall** | 0.10 (0.07 - 0.16) | 0.21 (0.13 - 0.39) | 0.47 (0.27 - 0.79) | 0.76 (0.48 - 0.98) | 0.08 (0.06 - 0.23) |
|  | **CAM** | 0.07 (0.06 - 0.10) | 0.13 (0.09 - 0.22) | 0.27 (0.17 - 0.50) | 0.49 (0.30 - 0.80) | 0.06 (0.05 - 0.11) |
|  | **Pharmacotherapy** | 0.10 (0.07 - 0.16) | 0.21 (0.13 - 0.40) | 0.48 (0.28 - 0.81) | 0.77 (0.50 - 0.98) | 0.09 (0.06 - 0.25) |
|  | **Psychotherapy** | 0.09 (0.07 - 0.13) | 0.18 (0.12 - 0.29) | 0.40 (0.24 - 0.65) | 0.67 (0.43 - 0.91) | 0.06 (0.05 - 0.09) |
| **Anxiety** | **Overall** | 0.10 (0.08 - 0.16) | 0.22 (0.14 - 0.41) | 0.49 (0.29 - 0.80) | 0.77 (0.51 - 0.98) | 0.08 (0.05 - 0.19) |
|  | **CAM** | 0.07 (0.07 - 0.09) | 0.13 (0.11 - 0.20) | 0.31 (0.22 - 0.47) | 0.58 (0.40 - 0.78) | 0.08 (0.05 - 0.14) |
|  | **Pharmacotherapy** | 0.11 (0.08 - 0.20) | 0.24 (0.15 - 0.50) | 0.55 (0.31 - 0.89) | 0.83 (0.54 - 0.99) | 0.11 (0.06 - 0.25) |
|  | **Psychotherapy** | 0.09 (0.07 - 0.13) | 0.18 (0.13 - 0.31) | 0.41 (0.26 - 0.68) | 0.67 (0.47 - 0.93) | 0.06 (0.05 - 0.10) |
| **Mood** | **Overall** | 0.11 (0.08 - 0.18) | 0.25 (0.15 - 0.46) | 0.58 (0.33 - 0.87) | 0.87 (0.59 - 0.99) | 0.09 (0.06 - 0.21) |
|  | **CAM** | 0.08 (0.07 - 0.13) | 0.16 (0.10 - 0.30) | 0.35 (0.20 - 0.61) | 0.58 (0.34 - 0.88) | 0.06 (0.05 - 0.10) |
|  | **Pharmacotherapy** | 0.12 (0.08 - 0.19) | 0.27 (0.16 - 0.47) | 0.61 (0.36 - 0.88) | 0.90 (0.62 - 0.99) | 0.09 (0.06 - 0.23) |
|  | **Psychotherapy** | 0.08 (0.07 - 0.11) | 0.15 (0.10 - 0.26) | 0.34 (0.19 - 0.56) | 0.58 (0.33 - 0.84) | 0.06 (0.05 - 0.09) |
| **Psychosis** | **Overall** | 0.09 (0.07 - 0.13) | 0.18 (0.11 - 0.32) | 0.40 (0.23 - 0.69) | 0.68 (0.43 - 0.94) | 0.08 (0.06 - 0.25) |
|  | **CAM** | 0.07 (0.06 - 0.09) | 0.10 (0.08 - 0.17) | 0.21 (0.14 - 0.39) | 0.42 (0.24 - 0.67) | 0.05 (0.05 - 0.11) |
|  | **Pharmacotherapy** | 0.09 (0.07 - 0.13) | 0.18 (0.11 - 0.32) | 0.41 (0.24 - 0.70) | 0.68 (0.43 - 0.94) | 0.09 (0.06 - 0.26) |
|  | **Psychotherapy** | 0.10 (0.08 - 0.13) | 0.22 (0.14 - 0.30) | 0.49 (0.32 - 0.65) | 0.76 (0.54 - 0.91) | 0.06 (0.05 - 0.09) |

*Table notes: CAM = complementary and alternative medicine. OR = odds ratio. ES_MA_ = meta-analytic effect size*

**Supplemental table 14: Power for each combination of experimental group and comparator for continuous safety outcomes.**

| **Experimental group** | **Comparator** | **Power** | |
| --- | --- | --- | --- |
|  |  | **SMD=0.40** | **ES_MA_** |
|  |  | **Median (IQR)** | **Median (IQR)** |
| Pharmacotherapy (mono) | Placebo | 0.84 (0.65 - 0.95) | 0.17 (0.07 - 0.90) |
|  | Pharmacotherapy (mono) | 0.75 (0.28 - 0.95) | 0.21 (0.08 - 0.73) |
| Combination pharmacotherapy | Pharmacotherapy (mono) | 0.91 (0.40 - 0.96) | 0.77 (0.20 - 1.00) |
| Psychotherapy | Any comparator | 0.43 (0.28 - 0.83) | 0.16 (0.09 - 0.37) |
| CAM | Any comparator | 0.37 (0.29 - 0.45) | 0.76 (0.33 - 0.98) |

*Table notes: CAM = complementary and alternative medicine. SMD = standardized mean difference. ES_MA_ = meta-analytic effect size.*

**Supplemental table 15: Power for each combination of experimental group and comparator for binary efficacy outcomes.**

| **Experimental group** | **Comparator** | **Power** | |
| --- | --- | --- | --- |
|  |  | **OR=2.0** | **ES_MA_** |
|  |  | **Median (IQR)** | **Median (IQR)** |
| Pharmacotherapy (mono) | Placebo | 0.26 (0.15 - 0.60) | 0.46 (0.22 - 0.82) |
|  | Pharmacotherapy (mono) | 0.26 (0.18 - 0.55) | 0.07 (0.06 - 0.14) |
| Combination pharmacotherapy | Pharmacotherapy (mono) | 0.20 (0.14 - 0.29) | 0.24 (0.12 - 0.40) |
| Pharmacotherapy + psychotherapy | Psychotherapy | 0.20 (0.16 - 0.31) | 0.12 (0.08 - 0.17) |
|  | Pharmacotherapy (mono) | 0.24 (0.15 - 0.39) | 0.23 (0.12 - 0.38) |
| Psychotherapy | Placebo/attention control | 0.23 (0.14 - 0.29) | 0.52 (0.29 - 0.65) |
|  | Treatment-as-usual/waitlist | 0.22 (0.16 - 0.31) | 0.54 (0.25 - 0.81) |
|  | Psychotherapy | 0.19 (0.12 - 0.26) | 0.08 (0.05 - 0.17) |
|  | Pharmacotherapy | 0.18 (0.16 - 0.24) | 0.09 (0.07 - 0.15) |
|  | Any comparator | 0.41 (0.22 - 0.56) | 0.50 (0.31 - 0.80) |
| CAM + regular treatment | Any active comparator | 0.23 (0.18 - 0.30) | 0.33 (0.08 - 0.68) |
| CAM | Placebo/treatment-as-usual/waitlist | 0.38 (0.19 - 0.56) | 0.49 (0.08 - 0.77) |
|  | Any active comparator | 0.38 (0.20 - 0.68) | 0.07 (0.06 - 0.14) |

*Table notes: CAM = complementary and alternative medicine. OR = odds ratio. ES_MA_ = meta-analytic effect size.*

**Supplemental table 16: Power for each combination of experimental group and comparator for binary safety outcomes.**

| **Experimental group** | **Comparator** | **Power** | |
| --- | --- | --- | --- |
|  |  | **OR=2.0** | **ES_MA_** |
|  |  | **Median (IQR)** | **Median (IQR)** |
| Pharmacotherapy (mono) | Placebo | 0.23 (0.13 - 0.46) | 0.10 (0.06 - 0.28) |
|  | Pharmacotherapy (mono) | 0.21 (0.13 - 0.39) | 0.09 (0.06 - 0.25) |
| Combination pharmacotherapy | Pharmacotherapy (mono) | 0.15 (0.10 - 0.26) | 0.06 (0.05 - 0.11) |
| Pharmacotherapy + psychotherapy | Psychotherapy | 0.16 (0.13 - 0.23) | 0.08 (0.06 - 0.16) |
|  | Pharmacotherapy (mono) | 0.18 (0.13 - 0.29) | 0.07 (0.06 - 0.10) |
| Psychotherapy | Placebo/attention control | 0.21 (0.15 - 0.24) | 0.05 (0.05 - 0.06) |
|  | Treatment-as-usual/waitlist | 0.17 (0.12 - 0.29) | 0.06 (0.05 - 0.09) |
|  | Psychotherapy | 0.18 (0.11 - 0.27) | 0.06 (0.05 - 0.09) |
|  | Pharmacotherapy | 0.20 (0.16 - 0.29) | 0.08 (0.06 - 0.11) |
|  | Any comparator | 0.19 (0.12 - 0.39) | 0.07 (0.05 - 0.11) |
| CAM + regular treatment | Pharmacotherapy (mono) | 0.10 (0.08 - 0.16) | 0.06 (0.05 - 0.13) |
| CAM | Placebo | 0.17 (0.11 - 0.29) | 0.06 (0.05 - 0.07) |
|  | Treatment-as-usual/waitlist | 0.09 (0.06 - 0.11) | 0.05 (0.05 - 0.06) |
|  | Any active comparator | 0.13 (0.10 - 0.31) | 0.10 (0.06 - 0.24) |

*Table notes: CAM = complementary and alternative medicine. OR = odds ratio. ES_MA_ = meta-analytic effect size.*

**Supplemental table 17: ES_avg_ for each intervention-comparator combination, for continuous efficacy outcomes only (sensitivity analyses)**

| **Experimental group** | **Comparator** | **Number of meta-analyses** | **ES_avg_** |
| --- | --- | --- | --- |
| Pharmacotherapy (mono) | Placebo | 611 | 0.33 |
|  | Pharmacotherapy (mono) | 689 | 0.15 |
| Combination pharmacotherapy | Pharmacotherapy (mono) | 120 | 0.31 |
| Pharmacotherapy + psychotherapy | Psychotherapy | 181 | 0.29 |
|  | Pharmacotherapy (mono) | 87 | 0.3 |
| Psychotherapy | Placebo/attention control | 29 | 0.18 |
|  | Treatment-as-usual/waitlist | 588 | 0.50 |
|  | Psychotherapy | 279 | 0.22 |
|  | Pharmacotherapy | 16 | 0.14 |
|  | Any comparator | 96 | 0.21 |
| CAM + regular treatment | Pharmacotherapy (mono) | 31 | 0.62 |
| CAM | Placebo | 46 | 0.26 |
|  | Treatment-as-usual/waitlist | 31 | 0.57 |
|  | Any active comparator | 39 | 0.22 |

*Table notes: ES_avg_ = meta-analytic average effect size of all meta-analyses for each combination of experimental group and comparator group.*

**Supplemental table 18: Median power by disorder and intervention category in sensitivity analyses, for continuous efficacy outcomes (sensitivity analyses)**

| **Disorder** | **Inter-vention** | **Power** | | |
| --- | --- | --- | --- | --- |
|  |  | **ES_avg_** | **ES_MA_**  **(meta-analyses with ES_MA_<0.20 excluded)** | **ES_MA_**  **(ES_MA_ based on the largest RCT in each meta-analysis)** |
|  |  | **Median (IQR)** | **Median (IQR)** | **Median (IQR)** |
| **Overall** | **Overall** | 0.20 (0.11 - 0.42) | 0.43 (0.22 - 0.76) | 0.17 (0.07 - 0.49) |
|  | **CAM** | 0.26 (0.13 - 0.49) | 0.48 (0.21 - 0.88) | 0.18 (0.07 - 0.66) |
|  | **Pharmacotherapy** | 0.14 (0.10 - 0.27) | 0.37 (0.18 - 0.71) | 0.16 (0.07 - 0.45) |
|  | **Psychotherapy** | 0.27 (0.14 - 0.48) | 0.47 (0.25 - 0.78) | 0.18 (0.08 - 0.53) |
| **Anxiety** | **Overall** | 0.24 (0.13 - 0.46) | 0.46 (0.24 - 0.78) | 0.23 (0.10 - 0.60) |
|  | **CAM** | 0.16 (0.14 - 0.62) | 0.31 (0.25 - 0.57) | 0.26 (0.21 - 0.53) |
|  | **Pharmacotherapy** | 0.17 (0.12 - 0.39) | 0.35 (0.18 - 0.69) | 0.26 (0.12 - 0.58) |
|  | **Psychotherapy** | 0.26 (0.15 - 0.46) | 0.49 (0.26 - 0.80) | 0.21 (0.09 - 0.61) |
| **Mood** | **Overall** | 0.20 (0.11 - 0.47) | 0.37 (0.19 - 0.69) | 0.13 (0.06 - 0.38) |
|  | **CAM** | 0.20 (0.12 - 0.38) | 0.42 (0.19 - 0.86) | 0.13 (0.06 - 0.48) |
|  | **Pharmacotherapy** | 0.17 (0.11 - 0.31) | 0.37 (0.20 - 0.67) | 0.15 (0.07 - 0.42) |
|  | **Psychotherapy** | 0.38 (0.13 - 0.88) | 0.35 (0.19 - 0.64) | 0.09 (0.05 - 0.28) |
| **Psychosis** | **Overall** | 0.11 (0.09 - 0.22) | 0.39 (0.18 - 0.81) | 0.10 (0.06 - 0.34) |
|  | **CAM** | 0.58 (0.48 - 0.70) | 0.63 (0.35 - 0.99) | 0.71 (0.15 - 0.96) |
|  | **Pharmacotherapy** | 0.10 (0.09 - 0.16) | 0.38 (0.18 - 0.80) | 0.09 (0.06 - 0.30) |
|  | **Psychotherapy** | 0.25 (0.13 - 0.46) | 0.33 (0.18 - 0.76) | 0.13 (0.06 - 0.34) |

*Table notes: Several sensitivity analyses were performed. First, we calculated power to detect the ES_avg_, the average meta-analytic effect size for each experimental group – comparator combination. Secondly, we calculated power to detect the ES_MA_ after exclusion of meta-analyses with an absolute effect size smaller than 0.20. Thirdly, we calculated power to detect the ES_MA_ based on the largest RCT in each meta-analysis, to reduce the influence of publication bias on the ES_MA_. CAM = complementary and alternative medicine*

**Supplemental table 19: Sensitivity analysis of median power for a range of effect sizes and the ES_MA_ by disorder and intervention category, for continuous efficacy outcomes, only including each study once.**

| **Disorder** | **Inter-vention** | **Power** | | | | |
| --- | --- | --- | --- | --- | --- | --- |
|  |  | **SMD=0.20** | **SMD=0.40** | **SMD=0.60** | **SMD=0.80** | **ES_MA_** |
|  |  | **Median (IQR)** | **Median (IQR)** | **Median (IQR)** | **Median (IQR)** | **Median (IQR)** |
| **Overall** | **Overall** | 0.12 (0.09 - 0.19) | 0.33 (0.20 - 0.58) | 0.63 (0.40 - 0.90) | 0.86 (0.62 - 0.99) | 0.20 (0.07 - 0.60) |
|  | **CAM** | 0.12 (0.09 - 0.15) | 0.32 (0.20 - 0.45) | 0.60 (0.38 - 0.79) | 0.84 (0.60 - 0.96) | 0.34 (0.16 - 0.74) |
|  | **Pharmacotherapy** | 0.13 (0.09 - 0.23) | 0.37 (0.23 - 0.69) | 0.68 (0.44 - 0.96) | 0.90 (0.68 - 1.00) | 0.12 (0.06 - 0.40) |
|  | **Psychotherapy** | 0.10 (0.08 - 0.15) | 0.27 (0.18 - 0.46) | 0.53 (0.35 - 0.79) | 0.77 (0.56 - 0.96) | 0.37 (0.12 - 0.79) |
| **Anxiety** | **Overall** | 0.10 (0.08 - 0.15) | 0.26 (0.18 - 0.45) | 0.50 (0.34 - 0.78) | 0.74 (0.55 - 0.96) | 0.42 (0.16 - 0.83) |
|  | **CAM** | 0.11 (0.09 - 0.12) | 0.31 (0.23 - 0.32) | 0.59 (0.46 - 0.61) | 0.83 (0.69 - 0.85) | 0.30 (0.23 - 0.40) |
|  | **Pharmacotherapy** | 0.12 (0.08 - 0.25) | 0.32 (0.18 - 0.72) | 0.61 (0.34 - 0.97) | 0.85 (0.55 - 1.00) | 0.29 (0.12 - 0.72) |
|  | **Psychotherapy** | 0.10 (0.08 - 0.13) | 0.24 (0.17 - 0.38) | 0.47 (0.33 - 0.69) | 0.71 (0.53 - 0.91) | 0.52 (0.19 - 0.87) |
| **Mood** | **Overall** | 0.13 (0.09 - 0.25) | 0.37 (0.21 - 0.73) | 0.68 (0.41 - 0.97) | 0.90 (0.63 - 1.00) | 0.15 (0.07 - 0.42) |
|  | **CAM** | 0.11 (0.08 - 0.16) | 0.30 (0.18 - 0.47) | 0.58 (0.33 - 0.80) | 0.82 (0.53 - 0.96) | 0.29 (0.14 - 0.63) |
|  | **Pharmacotherapy** | 0.14 (0.09 - 0.28) | 0.42 (0.23 - 0.78) | 0.76 (0.46 - 0.98) | 0.94 (0.69 - 1.00) | 0.12 (0.07 - 0.39) |
|  | **Psychotherapy** | 0.11 (0.08 - 0.22) | 0.32 (0.18 - 0.67) | 0.60 (0.35 - 0.95) | 0.84 (0.56 - 1.00) | 0.17 (0.08 - 0.41) |
| **Psychosis** | **Overall** | 0.12 (0.10 - 0.17) | 0.35 (0.24 - 0.52) | 0.66 (0.46 - 0.85) | 0.88 (0.70 - 0.98) | 0.11 (0.06 - 0.49) |
|  | **CAM** | 0.12 (0.10 - 0.14) | 0.33 (0.24 - 0.41) | 0.63 (0.47 - 0.73) | 0.86 (0.71 - 0.93) | 0.62 (0.34 - 0.98) |
|  | **Pharmacotherapy** | 0.12 (0.09 - 0.18) | 0.35 (0.23 - 0.54) | 0.66 (0.46 - 0.87) | 0.88 (0.69 - 0.98) | 0.09 (0.06 - 0.28) |
|  | **Psychotherapy** | 0.12 (0.10 - 0.16) | 0.35 (0.27 - 0.48) | 0.66 (0.53 - 0.82) | 0.88 (0.77 - 0.97) | 0.35 (0.12 - 0.86) |

*Table notes: CAM = complementary and alternative medicine. SMD = standardized mean difference. ES_MA_ = meta-analytic effect size.*
