# Supplementary figures and images for: Statistical power in clinical trials of interventions for mood, anxiety, and psychotic disorders
Source: Psychol Med. 2022 May 19;53(10):4499–506. doi: 10.1017/S0033291722001362 (PMC10388329; doi:10.1017/S0033291722001362)

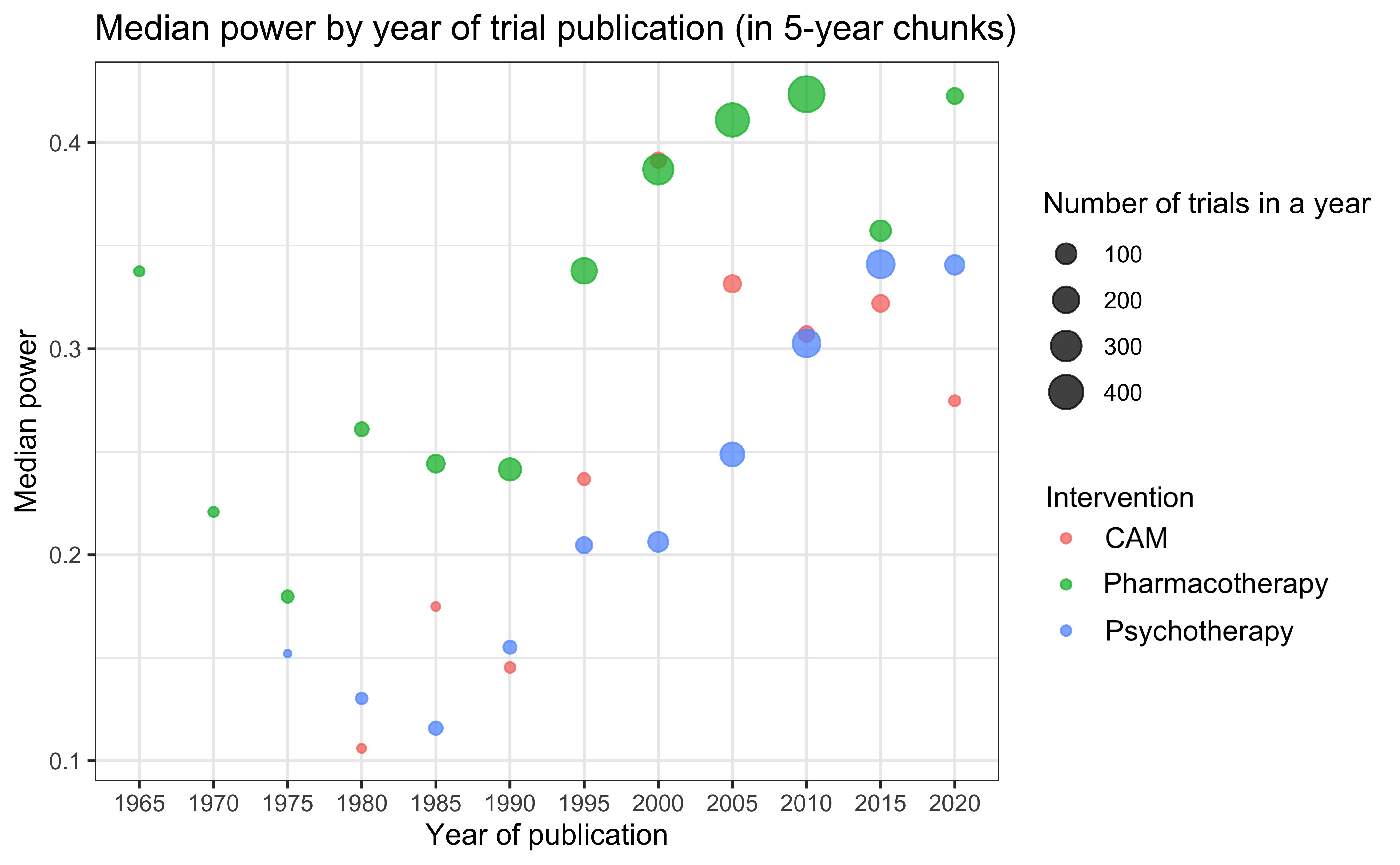

Supplement: Supplementary file 1 [file S0033291722001362sup.zip › S0033291722001362sup001.jpg]
